# Surrounding and background shades effect on color adjustment of single-shade composites

**DOI:** 10.1590/0103-6440202405742

**Published:** 2024-07-22

**Authors:** Márcia Luciana Carregosa Santana, Gabriella de Jess Santos Livi, Tauan Rosa Santana, Paula Fernanda Damasceno Silva, Clara Lemos Leal Barata de Matos, Carolina Menezes Maciel, André Luis Faria-e-Silva

**Affiliations:** 1. Graduate Program in Dentistry, Federal University of Sergipe, Aracaju, SE, Brazil; 2 Department of Dentistry, Federal University of Sergipe, Aracaju, SE, Brazil

**Keywords:** Color assessments, Color blending, Single-shade composite, Resin composite

## Abstract

This study evaluated the effects of surrounding and background shades on the color adjustment potential (CAP) and visual color match of two single-shade composites, Vittra APS Unique and Charisma Diamond One. Cylinder-shaped specimens were constructed, consisting of either single-shade composites alone (simple) or composites surrounded by the Forma material at shades A1 or A3 (dual). Simple specimens using only the Forma at the specified shades were also prepared. Color measurements of simple specimens were taken against a gray background using a spectrophotometer. For dual specimens, the color of the inner composite was measured against a gray or chromatic (the same shade as the outer composite) background. Color differences (ΔE00) between the single-shade composites and the A1/A3 composite were calculated. CAP was determined by comparing data from simple and dual specimens. Four experienced dentists scored the color match (perfect to unacceptable) for each specimen using a viewing booth illuminated by an illuminant D65. Data were analyzed with repeated-measures ANOVA and the Kruskal-Wallis test. The results showed that both single-shade composites showed the lowest color discrepancies when they were compared to A1 and A3. Using a chromatic background only significantly affected the CAP when the outer composite was A3. The visual analysis showed poor color matches between the single-shade and control chromatic composites, except for Charisma Diamond One surrounded by A1. In conclusion, the CAP values of the evaluated single-shade composites were impacted by both surrounding shade and background color, and the color match of these materials tended to be poor.

## Resumo

Este estudo avaliou os efeitos da cor do entorno e do fundo no potencial de ajuste de cor (PAC) e na correspondência visual de cor de dois compósitos monocromáticos, Vittra APS Unique e Charisma Diamond One. Amostras em formato cilíndrico foram criadas utilizando os compósitos monocromáticos e de cor A1/A3. Algumas amostras dos compósitos monocromáticos foram circundadas pelos compósitos A1 ou A3, formando amostras duplas. Medidas de cor das amostras simples foram obtidas em um fundo cinza utilizando um espectrofotômetro. Para as amostras duplas, a cor do compósito interno foi medida em um fundo cinza ou cromático (com a mesma cor do compósito externo). As diferenças de cor (ΔE_00_) entre os compósitos de monocromáticos e os compósitos A1/A3 foram calculadas. O PAC foi determinado comparando os dados das amostras simples e duplas. Quatro dentistas experientes avaliaram a correspondência de cor (de perfeita a inaceitável) para cada espécime utilizando uma cabine de visualização iluminada por uma iluminante D65. Dados foram analisados por ANOVA de medidas repetidas e teste de Kruskal-Wallis test. Os resultados mostraram que ambos os compósitos monocromáticos apresentaram as menores discrepâncias de cor quando comparados a A1 que em relação a A3. O uso de um fundo cromático afetou significativamente o PAC apenas quando o compósito externo era A3. A análise visual mostrou correspondência de cor fraca entre os compósitos monocromáticos e os compósitos cromáticos de controle, com exceção do Charisma Diamond One circundado por A1. Em conclusão, os valores de PAC dos compósitos monocromáticos avaliados foram impactados tanto pela cor do entorno quanto pela cor de fundo, e a correspondência visual de cor desses materiais tendeu a ser ruim.

## Introduction

Accurately selecting the right resin composite shade is essential for successful direct esthetic restorations ^1^. This requires precise color measurement and skillful blending of opaque and translucent composites to achieve optical characteristics that match the surrounding teeth ^2^ ^,^ ^3^ ^,^ ^4^. However, many composite brands use the VITA classical shade guide, which only covers a third of the diverse colors in human teeth ^5^. This means that the restoration material must be able to adapt to the color of the surrounding substrate to achieve a more refined color match.

Composites with enhanced color adjustment potentials (CAP) have been developed to address this challenge. These composites can be used to simplify the restoration process by allowing a single shade to be used to match the color of the surrounding teeth ^6^ ^,^ ^7^ ^,^ ^8^ ^,^ ^9^ ^,^ ^10^. The translucency of these composites allows them to reflect the color of the underlying and surrounding substrates, affecting the restoration's final color ^2^ ^,^ ^10^ ^,^ ^11^ ^,^ ^12^. However, the CAP of these composites may be reduced in practical situations where the palatal wall is missing, such as in class IV cavities or diastema closures. This is because the darkness of the oral cavity can affect the final color of the restoration, making it appear grayish ^13^. Additionally, applying single-shade composites to dentin with altered color may compromise the color match of the restoration ^4^ ^,^ ^14^ ^,^ ^15^. For this reason, some composite manufacturers recommend using an opaquer and a chromatic composite layer in these scenarios.

The role of using a chromatic composite in unfavorable clinical situations to improve the color-blending ability of single-shade composites is not yet clear. This is because there is a lack of studies that have assessed the impact of this factor using color visual analysis. The present study addressed this gap by evaluating how surrounding and background shades influence the CAP and visual color match of two single-shade composites. We hypothesized that neither the [1] surrounding composite shade nor the [2] background would significantly affect the CAP and visual color match of two single-shade composites.

## Materials and methods

### Experimental design

This study investigated three independent variables: "single-shade composite," "surrounding shade," and "background." Two single-shade composites, Charisma Diamond One (Kulzer Dental, Wertheim, Germany) and Vittra APS Unique (FGM, Joinvile, SC, Brazil), were examined. These single-shade composites were surrounded by another composite in either shade A1 or A3. The specimens' colors were evaluated against a gray or chromatic background with the same shade as the surrounding composite. Instrumental assessments analyzed color differences and CAP. Additionally, a visual assessment of the color match between the single-shade composites and the surrounded/control composite was conducted.

### Confection of specimens

Disc-shaped specimens (n=5) were used in this study. For single-shade specimens, either single-shade composites or a more chromatic composite (Forma, Ultradent, Indaiatuba, SP, Brazil) were placed within a metallic matrix with a 10-mm diameter and 2.0-mm depth. Chromatic composites with translucency equivalent to dentin were used. A light-curing unit with an irradiance of approximately 800 mW/cm² (Radii-Cal, SDI, Victoria, Australia) was positioned 2 mm from the matrix and used to light-cure the composites for 20 seconds.

We employed a matrix with a 16-mm internal diameter to construct dual specimens. Within this matrix, a metal cylinder of 10 mm diameter was placed at the center. Initially, the matrix was filled with 2mm of Forma composite at shade A1 or A3, which underwent 20 seconds of light curing. Subsequently, the central metal cylinder was lowered, creating a 10-mm diameter space that was filled with one of the single-shade composites (n=5). The composites were light-cured for 40 seconds, with the light-curing unit positioned 2 mm away from the matrix. The specimen preparation method followed the protocol established in a previous study ^7^. Final specimen refinement was accomplished using aluminum oxide discs (Sof-lex, 3M ESPE, St. Paul, USA).

### Instrumental Color Analysis

A spectrophotometer (SP60, X-Rite, Grand Rapids, MI, USA) in reflectance mode was utilized to assess the color of the inner composite. Measurements were taken with a 2° observer angle and illuminant D65. For single-shade specimens and the inner composite of dual specimens, measurements were conducted against a neutral gray background. Furthermore, chromatic backgrounds (16 mm in diameter and 2 mm in thickness) were prepared using Forma composite in shades A2 and A3. Consequently, the color of the inner composites in dual specimens was also measured over these chromatic backgrounds. A glycerin solution was used as a coupling agent between the specimen and the background for all evaluations. In this case, the background shade matched that of the surrounding composite. We recorded the color coordinates were L*, a*, and b*.

Using only the simple specimen, color differences between the control composites (shades A1 and A3) and the single-shade composite were calculated (ΔE_00_*_Simple_) using the following formula ^16^ ^.^ ^17^:

$$Equation\;1:\;∆E_{00}=\;\sqrt{{\left(\frac{∆L^{'}}{K_{L}S_{L}}\right)}^{2}+{{\left(\frac{∆C^{'}}{K_{C}S_{C}}\right)}^{2}+\;\left(\frac{∆H^{'}}{K_{H}S_{H}}\right)}^{2}+\;R_{T}\frac{∆C^{'}}{K_{C}S_{C}}\;\frac{∆H^{'}}{K_{H}S_{H}}}$$

Here, ΔL', ΔC', and ΔH' represent changes in luminosity, chroma, and hue, respectively. SL, SC, and SH denote weighted functions for each component, while KL, KC, and KH are weighted factors for Lightness, Chroma, and Hue (all set to 1) [17]. RT signifies the interactive term between chroma and hue differences. The same formula was employed to calculate color differences between the control simple specimens (A1 or A3) and the inner area of dual specimens with matching surrounding shades. This value was termed ΔE_00_*_Dual_. Finally, CAP values were determined using the following formula ^6^:

$$Equation\;2:\;CAP=1-({\Delta E}_{00}{*}_{Dual}/{\Delta E}_{00}{*}_{Simple})$$

### Visual Color Analysis

The visual color analysis took place in a controlled viewing booth, illuminated by four 30W lamps producing D65 illuminant with a Color Rendering Index (CRI) exceeding 90. The specimens were positioned on a sample holder inclined at 45° relative to the illuminating source. This task was undertaken by four dentists specialized in Restorative Dentistry or Prosthesis. The evaluators were tasked with assigning color similarity scores based on pairwise comparisons of simple specimens and determining the color match between the inner and outer composites in the dual specimens. Ratings were assigned on a scale of 1 to 5, representing the following gradations: 1−Perfect, 2−Very good, 3−Good, 4−Poor, and 5−Unacceptable.

The process involved assessing side-by-side comparisons of simple specimens of the single-shade composites alongside a chromatic composite specimen (shade A1 or A3), all presented against a consistent neutral gray background - the same background employed in the instrumental analysis. Furthermore, the dual specimens were observed against both the neutral gray background and the chromatic background matching the shade of the surrounding composite. The evaluations were blinded, and their sequence was randomized for each evaluator using the "random" function in Microsoft Excel 365.

### Data analysis

The Shapiro-Wilk test was used to assess the normality of the data for ΔE_00_ and CAP. Levene's test was used to verify the homogeneity of variance. The data for ΔE_00_*_Simple_ were analyzed using a two-way ANOVA, with the factors of "single-shade composite" and "control shade". Repeated measures ANOVA was used to analyze the data for ΔE_00_*_Dual_ and CAP, with the factors of "single-shade composite", "surrounding shade", and "background" (a repeated measure). For the visual color analysis, the Kruskal-Wallis test was used to compare median scores among the experimental conditions. All data analyses were performed using the open statistical platform Jamovi 1.6.15 (www.jamovi.org), with a confidence level of 95%.

## Results

The results of the ΔE_00_ measurements for the simple specimens are presented in Table 1. Two-way ANOVA revealed that the "single-shade composite" factor alone (p = 0.597) did not significantly affect color differences. However, the p-values for both the "control shade" (<0.001) and the interaction between the factors (0.001) were found to be significant. For both single-shade composites, it was observed that higher color discrepancies occurred with the control shade A3 compared to A1. No noticeable difference was observed between the single-shade composites when ΔE_00_ was calculated against composite A3. In contrast, when calculating ΔE_00_ against the control A1, higher values were noted for Charisma Diamond One compared to Vittra APS Unique.

**Table 1:** Mean and standard deviation of ΔE_00_ values for simple specimens according to single-shade composite and control shade (n = 5).

Single-shade composite	Control shade	
	A1	A3
Charisma Diamond One	4.91 (1.24) ^B^	8.49 (0.66) ^A^
Vittra APS Unique	3.45 (0.24) ^C^	9.60 (0.15) ^A^
		

Distinct letters indicate statistical differences among the pairwise comparisons at Tukey`s test (p < 0.05).

Table 2 displays the results of ΔE_00_ measurements for the dual specimens. Repeated measures ANOVA demonstrated that all examined factors (p<0.001 for all) affected the color difference values. Only the interaction between "single-shade composites" and "background" did not show significant influence (p=0.083), while the other double interactions (p<0.001) and the triple interactions (p<0.001) were found to be significant. The most pronounced color discrepancies emerged when comparing specimens with the surrounding shade A3 against the gray background. Disparities between the composites were solely evident for the surrounding shade A3, with Charisma Diamond One exhibiting the lowest ΔE_00_ values. Similarly, the background color significantly affected the ΔE_00_ values only for the surrounding shade A3, and a noticeable reduction was observed when a chromatic background was utilized.

**Table 2:** Mean and standard deviation of Δ_E00_ values for dual specimens according to single-shade composite, surrounding shade, and background used (n = 5).

Single-shade composite	Surrounding shade	Background	
		Gray	Chromatic^1^
Charisma Diamond One	A1	3.43 (0.68) ^D^	3.16 (0.28) ^D^
	A3	7.74 (0.17) ^B^	3.03 (0.53) ^D^
Vittra APS Unique	A1	3.77 (0.10) ^D^	3.03 (0.12) ^D^
	A3	9.96 (0.07) ^A^	6.37 (0.08) ^C^

Distinct letters indicate statistical differences among the pairwise comparisons at Tukey`s test (p < 0.05).

1. The same as the surrounding shade

Table 3 shows the results for CAP. Repeated measures ANOVA showed that all evaluated factors (p<0.001 for all) significantly influenced the CAP values. The only interaction that did not show a significant influence was the interaction between "single-shade composites" and "background" (p=0.762). However, the other double interactions (p<0.001) and the triple interactions (p<0.001) were found to be significant. In general, measuring the dual specimens' color against a chromatic background enhanced the CAP values. However, for Charisma Diamond One, significant differences between the backgrounds were only observed for the surrounding shade A3. Charisma Diamond One showed higher CAP values than Vittra APS Unique for all experimental conditions.

**Table 3:** Means and standard deviations of color adjustment potential (CAP) values by single-shade composite, surrounding shade, and background used (n = 5).

Single-shade composite	Surrounding shade	Background		
		Gray	Chromatic^1^
Charisma Diamond One	A1	0.30 (0.14) ^B^	0.36 (0.06) ^B^
	A3	0.09 (0.02) ^C^	0.64 (0.06) ^A^
Vittra APS Unique	A1	-0.09 (0.03) ^D^	0.12 (0.03) ^C^
	A3	-0.04 (0.01) ^D^	0.34 (0.01) ^B^

Distinct letters indicate statistical differences among the pairwise comparisons at Tukey`s test (p < 0.05).

1. The same as the surrounding shade.

The evaluation results for the restoration color match, as provided by the evaluators, have been tabulated in Table 4. Among the simple specimens, the Charisma Diamond One against the control composite A1 achieved the lowest color mismatch score; meanwhile, no notable disparity was observed in the other pairwise comparisons. Similarly, in the context of dual specimens, Charisma Diamond One surrounded by composite A1 garnered the highest color match scores, whereas the lowest scores were attributed to Vittra APS Unique surrounded by composite A3. When comparing dual specimens and their corresponding pairwise simple specimens, significant differences only emerged for Charisma Diamond One paired with the control or surrounding composite A3. Notably, in this experimental setting, assessing dual specimens against a chromatic background enhanced color matching.

**Table 4:** Medians and interquartile ranges of visual color adjustment scores for simple and dual specimens (n = 8).

Single-shade composite	Shade^1^	Simple specimens	Dual specimens		p-value
			Gray BG	Chromatic^2^ BG	
Charisma Diamond One	A1	2.00 (1.00) ^A^	2.00 (1.25) ^A^	1.50 (1.00) ^A^	0.835
	A3	5.00 (0.00) ^B^	4.00 (1.25) ^BC^	3.00 (0.00) ^B*^	0.016
Vittra APS Unique	A1	5.00 (1.25) ^B^	3.00 (2.00) ^AB^	3.00 (2.00) ^AB^	0.051
	A3	5.00 (0.00) ^B^	5.00 (0.00) ^C^	5.00 (0.25) ^C^	0.334
p-value		< 0.001	< 0.001	< 0.001	

The following scores were used: 1 - Perfect, 2 - Very good, 3 - Good, 4 - Poor, and 5 - unacceptable.

Distinct letters indicate statistical differences among Dwass-Steel-Critchlow-Fligner pairwise comparisons (p < 0.01).

1. Control shade for simple specimens and surrounding shade for dual specimens.

2. The same as the surrounding shade.

* Indicate statistical differences from the simple specimens.

BG: background.

## Discussion

The results of this study reveal several key points. When single-shade composites were surrounded by chromatic composites and measured against a gray background, color discrepancies were more pronounced for outer composite A3 than A1. However, changing the background to a chromatic one only impacted the color discrepancy for dual specimens surrounded by composite A3. The same trends were observed in CAP values, with the most significant changes occurring when the outer composite was A3. Notably, visual analysis indicated superior color matching when single-shade composites were encircled by chromatic composite A1, as opposed to A3. Thus, the first study`s hypothesis was rejected.

Furthermore, the study's findings suggest that CAP values measured against a chromatic background were consistently higher compared to those observed against a gray background, under the same experimental conditions (composite and surrounding shade). However, alterations in color match resulting from changes in background color were only significant for the single-shade composite Charisma Diamond One when surrounded by composite shade A3. Consequently, we also failed to reject the second study`s hypothesis.

When examining simple specimens, noticeably reduced color differences were apparent between both single-shade composites and the control composite in shade A1, as opposed to A3. Disregarding any potential influence from a neutral background, it is anticipated that the color of the evaluated single-shade composites in simple specimens would closely approximate their true color. This is because no discernible impact from the surrounding or more chromatic background on the specimens is expected. In fact, both single-shade composites appear whiter than both control composites, with color differences that can exceed two-fold (for A1) and five-fold (for A3) the 50:50% acceptability threshold (1.8) set in a previous study ^1^. Consequently, achieving an acceptable color match using these simplified composites relies on their capacity for color adjustment to both the surrounding and underlying substrate colors.

Only surrounding the single-shade composite Charisma Diamond One with either composite A1 or A3 yielded a slight reduction in color discrepancy for the single specimens constructed using these chromatic composites. Conversely, no discernible impact was noticed for the Vittra APS Unique composite. These findings underscore that only Charisma Diamond One composite exhibited the ability to adapt its color to match its surroundings, even though the changes in ΔE_00_ values (ranging from 0.75 to 1.48) might be of marginal clinical significance. Supporting this observation, the visual analysis failed to identify noteworthy differences in color matching when comparing simple specimens side by side or when contrasting the corresponding dual specimens placed against a gray background. An explanation is that color adjustment of the single-shade composites relies strongly on their pronounced translucency, allowing them to emulate the surrounding color ^8^ ^,^ ^9^. However, this mirroring effect becomes less pronounced towards the center of the restoration, which accounts for the minimal effect observed in CAP values by simply surrounding the composite with a more chromatic one ^18^.

Notably, introducing a chromatic background with a matching shade to the control composite led to a reduction in ΔE_00_ values, though this effect reached significance only for shade A3. Indeed, evaluating CAP values measured against the chromatic backgrounds highlighted that values for shade A3 were 78% higher (increasing from 0.36 to 0.64) for Charisma Diamond One composites and 183% higher (increasing from 0.12 to 0.34) for Vittra APS Unique composites compared to shade A1. However, it's crucial to emphasize that even when dual specimens were placed over a chromatic background, the lowest mean ΔE_00_ value observed was 3.03, surpassing the 50:50% acceptability threshold determined in a previous study ^1^.

Upon reviewing the outcomes of the visual analysis, superior scores emerged for color matching against the surrounding shade A1 compared to A3. Interestingly, altering the background color showed no notable impact on the results. Intriguingly, only Charisma Diamond One received a score of "very good" or better. However, similar scores were also noted when comparing simple Charisma Diamond One specimens with control composite A1 specimens side by side. This implies that positioning the latter composites adjacent to or around Charisma Diamond One had no discernible effect on color matching. A substantial enhancement in results was exclusively observed for dual specimens when assessing the color discrepancies between Charisma Diamond One and composite A3. This situation prompted a shift from "unacceptable" to "good" color matching by incorporating composite A3 around and beneath the evaluated single-shade composite. Another noteworthy observation is that Vittra APS Unique exhibited a color mismatch for composite A3, which was deemed "unacceptable," even when observed with dual specimens against a shaded A3 background. Intriguingly, the composite showing superior color adjustment (Charisma Diamond One) is specifically recommended for restorations in posterior teeth. In contrast, the manufacturer of Vittra APS Unique, despite its color mismatch, recommends its use for restorations in anterior teeth.

Although instrumental color analysis is validated and less prone to bias, visual analysis remains crucial in dental color evaluations due to its potential for yielding clinically relevant outcomes ^10^ ^,^ ^19^. In our study design, we made efforts to mitigate certain outcome biases by employing blinding procedures for evaluations and randomizing the evaluation sequence. Our findings indicate that even when utilized on favorable substrates or when incorporating an underlying chromatic resin layer, the capacity of the evaluated single-shade composites to adapt their color to the substrates appears to be markedly limited. Consequently, achieving imperceptible restorations using these materials may be challenging, especially when dealing with darker substrates.

A limitation of our study is that only four experienced dentists were involved in scoring the color matches. Therefore, it is plausible that different outcomes could be observed with less experienced clinicians or laypersons, who often apply less stringent evaluations. Another limitation of this study is the use of homogeneous composites as surrounds, which neglects the inherent color complexity of natural teeth. This could lead to discrepancies in color adjustment when translating our findings into clinical practice. Ultimately, the water sorption of composites has been shown to impact both their translucency ^19^, which can also affect their ability to achieve color adjustment. However, this aspect was not examined in the current study. Consequently, future investigations that involve tooth substrates as surroundings and incorporate water storage become essential to a more comprehensive understanding, offering clinically relevant insights for dental applications.

In conclusion, this study has revealed that the examined single-shade composites exhibit a limited capacity to harmonize their color with the adjacent substrate, especially when the background displays an unfavorable color (as observed with gray in the present study). The color adaptation of these materials is notably contingent on the underlying substrate color, emphasizing the importance of aligning it with that of the adjacent substrate.
